# Supplementary material for: Replacement of native by non-native animal communities assisted by human introduction and management on Isla Victoria, Nahuel Huapi National Park
Source: PeerJ. 2015 Oct 20;3:e1328. doi: 10.7717/peerj.1328 (PMC4662593; doi:10.7717/peerj.1328)
Supplement: Appendix S1 — Models fitted for Pheasant, Chucao, Deer and Boar and their corresponding AIC values. Functions: half-normal (hn), hazard-rate (haz), exponential (exp); detection covariates: type of environment (Env); density covariates: level of disturbance (Dis). [file peerj-03-1328-s001.docx]

Appendix S1: Model selection

Models fitted for Pheasant, Chucao, Deer and Boar and their corresponding AIC values. Functions: half-normal (hn), hazard-rate (haz), exponential (exp); detection covariates: type of environment (Env); density covariates: level of disturbance (Dis).

Pheasant

| \|  \| \| --- \| | nPars | AIC | delta | AICwt | cumltvWt |
| --- | --- | --- | --- | --- | --- | --- |
| haz_Dist | 4 | 111.52 | 0.00 | 4.0e-01 | 0.40 |
| haz_EnvDist | 5 | 111.53 | 0.01 | 4.0e-01 | 0.81 |
| exp_EnvDist | 4 | 113.44 | 1.92 | 1.5e-01 | 0.96 |
| exp_Dist | 3 | 116.40 | 4.89 | 3.5e-02 | 1.00 |
| haz | 3 | 121.33 | 9.81 | 3.0e-03 | 1.00 |
| hn_EnvDist | 4 | 123.57 | 12.05 | 9.8e-04 | 1.00 |
| hn_Dist | 3 | 126.43 | 14.91 | 2.3e-04 | 1.00 |
| hn | 2 | 136.24 | 24.72 | 1.7e-06 | 1.00 |
| exp | 2 | 183.53 | 72.01 | 9.3e-17 | 1.00 |

Chucao

| \|  \| \| --- \| | nPars | AIC | delta | AICwt | cumltvWt |
| --- | --- | --- | --- | --- | --- | --- |
| hn_EnvDist | 4 | 128.32 | 0.00 | 4.9e-01 | 0.49 |
| haz_Dist | 4 | 130.22 | 1.90 | 1.9e-01 | 0.68 |
| haz_EnvDist | 5 | 130.63 | 2.31 | 1.5e-01 | 0.83 |
| exp_EnvDist | 4 | 131.63 | 3.31 | 9.4e-02 | 0.93 |
| haz | 3 | 133.37 | 5.05 | 3.9e-02 | 0.97 |
| exp_Dist | 3 | 134.09 | 5.78 | 2.7e-02 | 0.99 |
| exp | 2 | 137.25 | 8.93 | 5.6e-03 | 1.00 |
| hn_Dist | 3 | 144.52 | 16.20 | 1.5e-04 | 1.00 |
| hn | 2 | 147.68 | 19.36 | 3.1e-05 | 1.00 |

Deer

| \|  \| \| --- \| | nPars | AIC | delta | AICwt | cumltvWt |
| --- | --- | --- | --- | --- | --- | --- |
| hn | 2 | 85.32 | 0.00 | 0.5187 | 0.52 |
| haz | 3 | 85.48 | 0.16 | 0.4799 | 1.00 |
| exp | 2 | 97.13 | 11.81 | 0.0014 | 1.00 |

Boar

| \|  \| \| --- \| | nPars | AIC | delta | AICwt | cumltvWt |
| --- | --- | --- | --- | --- | --- | --- |
| exp | 2 | 48.04 | 0.00 | 0.455 | 0.45 |
| haz | 3 | 48.05 | 0.00 | 0.452 | 0.91 |
| hn | 2 | 51.22 | 3.17 | 0.093 | 1.00 |
